# Supplementary material for: Gibberellin Signaling Promotes the Secondary Growth of Storage Roots in Panax ginseng
Source: Int J Mol Sci. 2021 Aug 13;22(16):8694. doi: 10.3390/ijms22168694 (PMC8395461; doi:10.3390/ijms22168694)
Supplement: Supplementary file 1 [file ijms-22-08694-s001.zip › Supplementary Figures.pdf]

Supplementary Figure S1

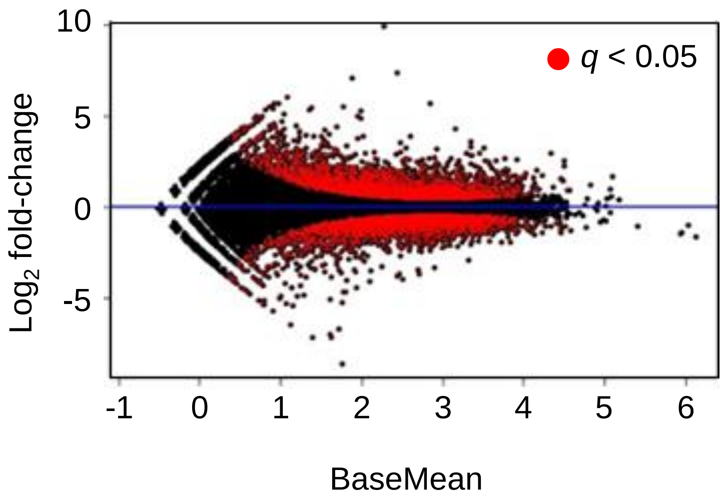

Figure S1. MA plot of differential expression between DMSO- and GA-treated samples. Red dots represent the either up- (2,805) and down- (2,916) regulated genes with $q < 0.05$ and ≥ |1.5|-fold change.

Supplementary Figure S2

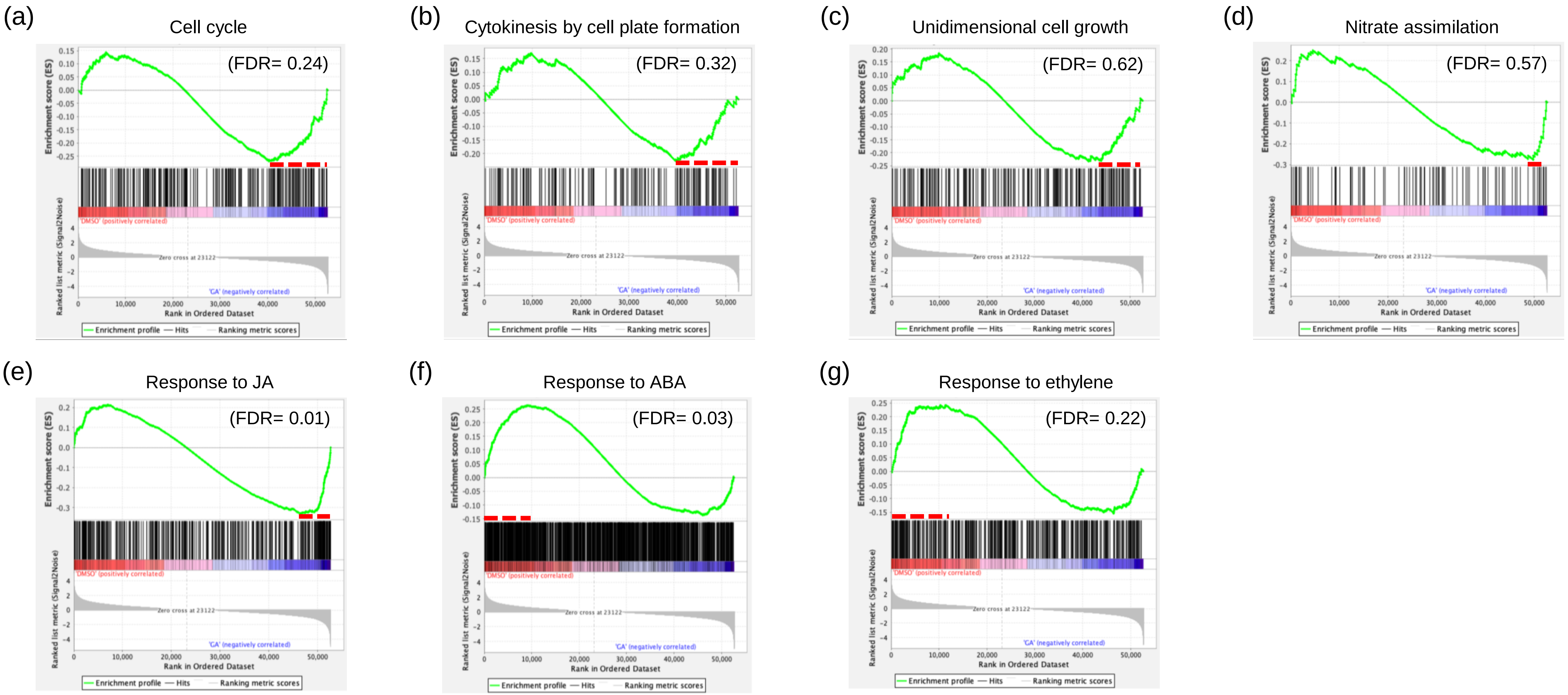

Figure S2. Enrichment plot for cell cycle, cytokinesis, cell growth, nitrate assimilation, and response to plant hormones using GSEA. In the plot, red dot line (----) indicates a leading-edge subset of the enriched gene set group leading to enrichment scores with respect to expression changes.

Supplementary Figure S3

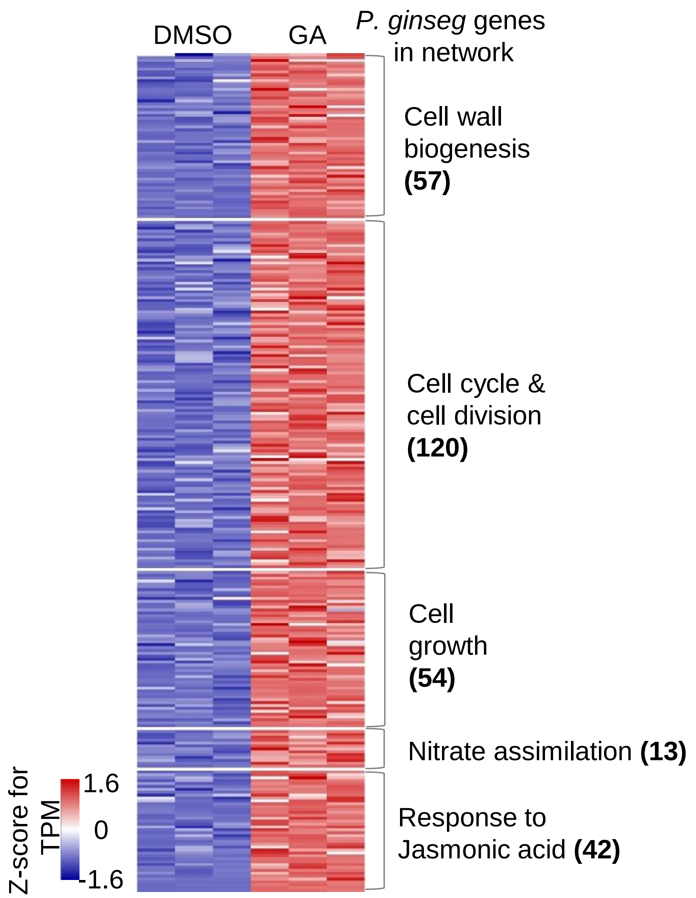

Figure S3. Expression heatmap of up-regulated genes contained in GA-induced transcriptional network.
