# Supplementary material for: Gibberellin Signaling Promotes the Secondary Growth of Storage Roots in Panax ginseng
Source: Int J Mol Sci. 2021 Aug 13;22(16):8694. doi: 10.3390/ijms22168694 (PMC8395461; doi:10.3390/ijms22168694)
Supplement: Supplementary file 1 [file ijms-22-08694-s001.zip › Supplementary Table S5.pdf]

Supplementary Table S5 . Primer combinations for gene cloning

Gene name	Sequences (5` to 3`)
GID1A	F : CGGGATCCATGGCTGGAAGTAGTGGTATTA
	R : AAGGCCTACAGTCAGAACCCACAAAGC
GID1B	F : CGGGATCCATGGCTGGAAGTAATGGAATTAATC
	R : AAGGCCTACAGTCAGAACCCACAAAGC
GID1C	F : AGATCTATGGCTGGGAGTAATGAAATTAATC
	R : AGGCCTGTAGGAACATACAAAACTTTTTATCTC
GID1D	F : GGATCCATGGCTGGTAGTAATGAAGTTAACG
	R : CCCGGGACAGTTAGGATTCACAAAGTTTTTTA
RGA1	F : AGATCTATGAAGAGAGACTACCCACAACCTAA
	R : AAGGCCTACCACTGAGTTTCCACGC
RGA2	F : CGGGATCCATGAAGCGAGACTATCCACAAC
	R : AAGGCCTACCACTGAGTTTCCAGGCTG
RGA3	F : AGATCTATGAAGAGAGACTACCCACAGC
	R : AGGCCTACCACTGAGTTTCCACGC
RGA4	F : AGATCTATGGTTATTAAGAGAGATCGCGAGC
	R : AGTACTCGGAGCTTCAGCAGCG
RGA5	F : AGATCTATGGTTATTAAGAGAGATCGCGAGC
	R : AGTACTCGGAGCTTCAGCAGCG
